# The way in which a physiotherapy service is structured can improve patient outcome from a surgical intensive care: a controlled clinical trial

**DOI:** 10.1186/cc11894

**Published:** 2012-12-11

**Authors:** Susan D Hanekom, Quinette Louw, Andre Coetzee

**Affiliations:** 1 Department of Interdisciplinary Health Sciences, Division of Physiotherapy, Stellenbosch University, Franci van Zyl Road, 7505 Tygerberg, South Africa; 2 Department of Anesthesiology and Critical Care, Stellenbosch University, Franci van Zyl Road, 7505 Tygerberg, South Africa

## Abstract

**Introduction:**

The physiological basis of physiotherapeutic interventions used in intensive care has been established. We must determine the optimal service approach that will result in improved patient outcome. The aim of this article is to report on the estimated effect of providing a physiotherapy service consisting of an exclusively allocated physiotherapist providing evidence-based/protocol care, compared with usual care on patient outcomes.

**Methods:**

An exploratory, controlled, pragmatic, sequential-time-block clinical trial was conducted in the surgical unit of a tertiary hospital in South Africa. Protocol care (3 weeks) and usual care (3 weeks) was provided consecutively for two 6-week intervention periods. Each intervention period was followed by a washout period. The physiotherapy care provided was based on the unit admission date. Data were analyzed with Statistica in consultation with a statistician. Where indicated, relative risks with 95% confidence intervals (CIs) are reported. Significant differences between groups or across time are reported at the alpha level of 0.05. All reported *P *values are two-sided.

**Results:**

Data of 193 admissions were analyzed. No difference was noted between the two patient groups at baseline. Patients admitted to the unit during protocol care were less likely to be intubated after unit admission (RR, 0.16; 95% CI, 0.07 to 0.71; RRR, 0.84; NNT, 5.02; *P *= 0.005) or to fail an extubation (RR, 0.23; 95% CI, 0.05 to 0.98; RRR, 0.77; NNT, 6.95; *P *= 0.04). The mean difference in the cumulative daily unit TISS-28 score during the two intervention periods was 1.99 (95% CI, 0.65 to 3.35) TISS-28 units (*P *= 0.04). Protocol-care patients were discharged from the hospital 4 days earlier than usual-care patients (*P *= 0.05). A tendency noted for more patients to reach independence in the transfers (*P *= 0.07) and mobility (*P *= 0.09) categories of the Barthel Index.

**Conclusions:**

A physiotherapy service approach that includes an exclusively allocated physiotherapist providing evidence-based/protocol care that addresses pulmonary dysfunction and promotes early mobility improves patient outcome. This could be a more cost-effective service approach to care than is usual care. This information can now be considered by administrators in the management of scarce physiotherapy resources and by researchers in the planning of a multicenter randomized controlled trial.

**Trial registration:**

PACTR201206000389290

## Introduction

Quality healthcare is defined as "the degree to which health services for individuals and populations increase the likelihood of desired health outcomes and are consistent with current professional knowledge" [1]. Although the physiological basis of many physiotherapeutic interventions used in intensive care units (ICUs) have been established, we need to determine the optimal service approach to improve patient outcome [2]. Surveys report variation in service approaches between countries, regions, and across individual units [3-5]. This variation is related to how the service is provided and which tasks are performed. Staffing levels, physiotherapists' training and expertise, physician referral patterns, and a perceived lack of benefit have been linked to this practice variation [3,4] Variations in practice of fellow ICU interdisciplinary team members have been linked to less optimal patient outcomes and increased cost [6].

Health administrators, faced with the economic realities of providing quality health care to increasing populations, are demanding measurement and accountability from professionals offering ICU services [7]. The European Society of Intensive Care Medicine Working Group on quality improvement recommends that a physiotherapist with expertise in the management of critically ill patients be available to a unit 7 days per week [8]. Recent work has highlighted the role of physiotherapists in facilitating early mobility of critically ill patients [9-12]. However, early mobility does not fully reflect the current evidence base of physiotherapy in the ICU [2]. The burden of proof is on the physiotherapy profession to find ways to quantify the value and to describe a quality physiotherapy service in the ICU setting [13].

Professions working in the ICU environment have developed specific outcomes to provide evidence of quality and benefit. High-intensity physician staffing levels have been linked to decreased ICU mortality, hospital and ICU length of stay, medical care costs, increased survival rates, and improved quality of dying [14]. An adequate nurse-to-patient ratio has been associated with decreased nosocomial infections [15], medication errors, and patient/family complaints [16]. Including a pharmacist in the interdisciplinary team is linked to decreased adverse drug events and cost of care [17]. Standards regulating qualifications of physicians and nurses working in this environment have been established [18]. We are unaware of any standards regulating physiotherapists' qualifications in the ICU [2]. In addition, physiotherapy-sensitive outcomes are currently lacking in the ICU environment [19].

### Preliminary work

**Figure 1:**
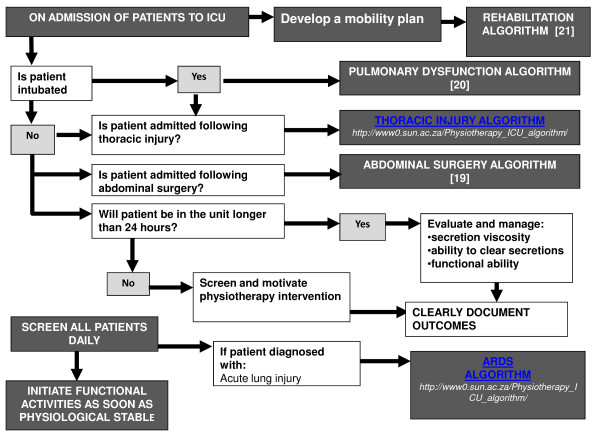
**Flowchart to direct algorithm use**.

This study was motivated by the lack of information available to guide the organization of physiotherapy services that would ensure optimal outcome for surgical ICU patients. The development and implementation of protocols based on best available evidence have been advocated to address practice variation [20], facilitate clinical decision making [21], and optimize evidence utilization by practitioners [22]. We developed an evidence-based protocol consisting of five clinical-management algorithms. This protocol was validated by a group of 27 national and international experts. The protocol addressed pulmonary dysfunction, muscle weakness, and functional insufficiencies in the surgical population (Figure 1) [23-25]. We reported in an earlier article that the implementation of the evidence-based physiotherapy protocol resulted in a physiotherapy service that was significantly different from usual care (Table 1) [26]. The aim of this article is to report on the estimated effect of providing a physiotherapy service consisting of an exclusively allocated physiotherapist providing evidence-based/protocol care, compared with usual care on patient outcomes.

**Table 1:** A comparison of the physiotherapy service provided during usual-care and protocol-care condition periods

	Usual care	Protocol care	*P*
Organization of service			

Percentage of therapy sessions provided over a weekend	10%	21%	<0.001

Rate of therapy sessions/patient/ICU day	0.57	1.38	<0.001

Duration of individual therapy session in minutes (mean ± SD)	23 ± 7	22 ± 11	0.94

Time (hours) from unit admission to first contact with therapist (mean ± SD)	27 ± 20	14 ± 7	<0.001

Content of sessions			

Percentage of therapy sessions that included techniques to remove bronchial secretions (suction; cough)	75%	35%	<0.001

Percentage of therapy sessions that included deep-breathing exercises	16%	34%	<0.001

Percentage of therapy sessions that included techniques to mobilize patient (passive/active/away from bed)	66%	82%	<0.001

Percentage of therapy sessions that included techniques to mobilize bronchial secretions (manual techniques; manual hyperinflation; vibromat)	52%	10%	<0.001

Percentage of therapy sessions that included techniques to improve pulmonary volumes (DBE; IPPB; PEP; recruitment)	39%	39%	0.96

Inclusion of rehabilitation management option; Odds ratio, 95% CI	2.34 (1.66 to 3.43)		<0.001

Inclusion of chest physiotherapy management option; Odds ratio, 95% CI	0.14 (0.09 to 0.22)		<0.001

CI, confidence interval; DBE, deep-breathing exercise; IPPB, intermittent positive-pressure breathing; PEP, positive expiratory pressure; SD, standard deviation.

## Materials and methods

### Trial design

Exploratory, controlled, pragmatic, sequential-time-block clinical trial. Two 6-week trial periods subdivided into four 3-week condition periods (Table 2). Each trial period was followed by a washout period to limit selection bias, as no elective surgery was scheduled (Table 2).

**Table 2:** Trial design

Intervention period	Time block	Care provided	Time period	Condition period
One	One	Usual care	1 to 21 November 2008	Usual-care condition period

One	Two	Protocol care	22 November to 12 December 2008	Protocol-care condition period

Washout period	Three	Usual care	13 December 2008 to 4 January 2009	Washout period Daily patient data were not collected. No new patient admissions into project

Two	Four	Usual care	5 to 25 January 2009	Usual-care condition period

Two	Five	Protocol care	26 January to 15 February 2009	Protocol-care condition period

Washout period	Six	Usual care	16 February 2009 until all trial patients had been discharged from the unit	Washout period Daily patient data were not collected. No new patient admissions into project

### Research setting

The 10-bed level-three closed surgical ICUs are situated in a tertiary hospital (1,385 beds) in South Africa. A surgical unit is one of seven specialized ICUs. All patients requiring support/monitoring, after elective/emergency surgery/trauma, are admitted to this unit, from theater/internal wards/resuscitation unit. A permanent matron is present and a 1:1.7 nurse-to- patient ratio [27]. A dietician is on call, and a medical technician is permanently allocated to the unit.

### Ethical considerations

The project is registered with institutional Research Ethics Committee (Project number 2003/055/N). This trial compared two different physiotherapy service delivery models. No new experimental procedures were introduced. Standard measures for identification and management of any adverse event as a result of physiotherapy intervention were in place. Proxy consent was obtained from the superintendent for all patients admitted to the unit during the trial period [28].

### Research team

The principal investigator (PI) ensured protocol standardization. Four nonspecialized therapists were recruited and appointed as *locum tenens *to the unit for the study duration [29]. These research therapists provided protocol care. Two ICU-specialized nursing practitioners (data assistants), were appointed to extract data from existing documentation systems. Two qualified physiotherapists (testing assistants) completed functional assessments within 48 hours of unit discharge.

#### Sample of convenience

All patients admitted to the surgical ICU consecutively during two trial periods, 1 November to 12 December 2008 and again from 5 January to 15 February 2009 were included. Patients were excluded if younger than 16 years and already in the unit on 1 November 2008 and 5 January 2009. Physiotherapy care provided was based on the unit admission date (Table 2).

Usual care was provided by the hospital physiotherapy department. One therapist is allocated to the 10-bed-unit per 3-month clinical cycle. Additional responsibilities include service to two 30-bed surgical inpatient wards. One morning per week, the amputation outpatient clinic is staffed by the therapist. During weekdays, the department provides an 8-hour on-site service to the hospital, and patients in ICU are managed routinely on a nonreferral basis. Over weekends, intensivists are limited to the referral of four ICU patients. In addition, a 24-hour/day; 7- day/week off-site on-call service is provided by all full-time staff members on a rotational basis. Patients are assessed, and the physiotherapeutic management is based on the therapists' clinical decision.

Evidence-based protocol-care: was provided to the unit by research therapists. Therapists were exclusively responsible for patient care in the 10-bed surgical ICU. A therapist was on-site for 12 hours during the week and 8 hours over a weekend. The research therapists worked in shifts. On-site shifts were limited to 8 hours per therapist. Off-site on-call service was offered by research therapists on a rotational basis. All patients in the unit were assessed daily by a research therapist and clinical decisions were guided by a flowchart (Figure 1) [26].

#### Standardization of care

Hospital therapists were at liberty to provide *appropriate *care based on patient assessment and expected outcome. No attempts were made to standardize the treatment offered, control the quality of the interventions used or limit the frequency of treatment sessions offered by hospital therapists.

Before trial commencement, research therapists were provided with protocol documentation and attended a 1-day workshop facilitated by PI. Research therapists' protocol adherence was monitored (PI) only during the first week of protocol care. No additional attempts were made to control the therapy (quality, frequency, or volume) provided.

Outcomes measured included the three categories of outcomes recognized in the ICU environment [30].

Clinical outcomes: Ventilation proportions*: *number of intubated patients in a condition period per number of patients admitted during that condition period.

Proportion of failed extubations: Number of failed extubations (patient reintubated 24 hours after extubation [31]) per number of extubations within a condition period.

Time on the ventilator: time (hours) from intubation to extubation a patient spent on the ventilator during the stay in the unit. Calculated as a sum of individual ventilation episodes: if ventilated on unit admission, admission time was used as intubation time (because of incomplete patient records previously reported [27]); if reintubated within 1 hour, the hour was regarded as ventilated time; when reintubation time exceeded 1 hour, individual ventilation duration per episode was calculated.

Time on ventilator was calculated for all patients admitted and discharged in a single intervention period. Unit and hospital mortality were reported. Economic outcomes: ICU length of stay (LOS) was calculated in hours from admission to unit discharge/death.

Hospital LOS was calculated for two periods: (1) Post-ICU LOS: from unit discharge to hospital discharge/death; and (2) hospital LOS: from hospital admission to discharge/death. The Therapeutic Intervention Scoring System (TISS-28) has been validated in surgical ICU and is used to determine nursing workload and as proxy for cost [32-35]. The value of a TISS-28 point ranges between 35 and 39.9 Euro [35-37]. It consists of a 28-item list of nursing activities [38]. The TISS-28 unit-day score was calculated daily for each patient remaining in the unit for the duration of a TISS-28 unit day. A TISS-28 unit-day was defined as the 24-hour period between 07:00 and 06:59 the following day.

### Patient centred outcomes

Function was evaluated within 48 hours of unit discharge by using the Barthel Index. All patients admitted and discharged in a single intervention period were eligible for functional testing. The Barthel Index (BI) measures a patient's perception of the capacity to execute 10 basic activities of daily living and gives a quantitative estimation of the patient's level of dependency, with scoring from 0 (totally dependent) to 100 (totally independent). The scale is valid and reliable and has been used in ICU populations [39,40].

Intervention and control groups were compared at baseline with regard to age, gender, admission diagnosis, severity of illness (APACHE II score), infective status, pre-unit LOS, and intubation status on admission.

### Data collection

Data were extracted from unit documents by data assistants by using standardized data-extraction forms and TISS-28 data sheets. The data-extraction process was standardized *a priori *to ensure data integrity. Testing assistants screened all patients before administering functional tests. The interpretation of the Barthel Index information was standardized *a priori*. Patients were instructed to answer questions pertaining to their function since unit discharge. Testing assistants were trained *a priori *to ensure interrater reliability of data.

### Controlling for contamination of the blinding process

Patients were blind to trial intervention and outcomes. Data and testing assistants were blind to condition period. Medical staff and therapists were blind to outcomes. Data were analyzed by a statistician blinded at the level of condition-period allocation.

### Data processing and statistical analysis

Data were analyzed with Statistica software, version nine, by Statsoft (Southern Africa Research (Pty) (Ltd)) in consultation with a statistician. Data were analyzed for each day of the admission-condition period. Central tendencies and data variability were reported as means/standard deviations if data were distributed normally; and medians/interquartile ranges when not. For continuous variables, Student *t *test, ANOVA, or repeated measures ANOVA was used to compare the groups. For categoric variables, the χ^2 ^or Fisher Exact test was used (as indicated). Mean differences between groups are reported with 95% CI of the mean. Where indicated, odds ratios; relative risks, with 95% CI and number needed to treat, are reported. A mixed-effect linear-regression model was used to investigate the activities of the TISS-28 unit-day scores over time, with the patient as the random effect and the fixed effect as period, and day in ICU, and the interaction. Significant differences between groups or across time are reported at the alpha level of 0.05. All reported *P *values are two-sided.

## Results

**Figure 2:**
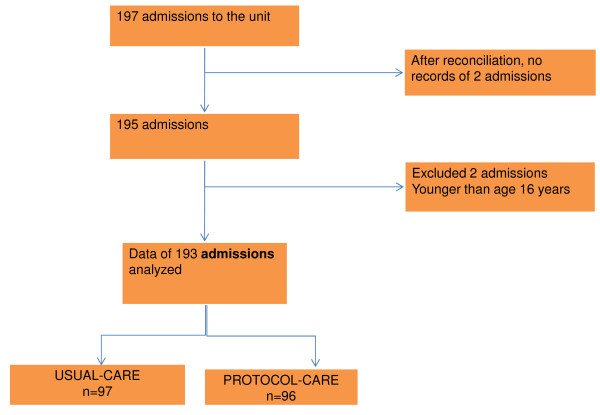
**Consort diagram of condition-period data analyzed**.

One hundred ninety-seven admissions to the unit were recorded. Data of 193 admissions were analyzed (Figure 2). No differences were noted at baseline (Table 3).

**Table 3:** Baseline comparison of protocol-care and usual-care groups

	Usual care *n *= 97	Protocol care *n *= 96	P value
Age, mean (SD)	50.18 (17.86)	52.07 (18.51)	0.47

APACHE, mean (SD)	16.24 (22.65)	18.40 (27.43)	0.55

Gender %M	60 (62%)	59 (61%)	0.97

Intubated on admission to the unit *n *(%)	55 (57%)	45 (47%)	0.22

Infective status on admission: all three criteria *n *(%)	8 (8%)	5 (5%)	0.40

Infective status on admission: at least one criterion *n *(%)	77 (79%)	81 (84%)	0.37

Infective status on admission: no criteria *n *(%)	5 (5%)	3 (3%)	0.48

Admission diagnosis: elective surgery *n *(%)	55 (57%)	55 (57%)	0.93

Admission diagnosis: emergency surgery *n *(%)	15 (15%)	14 (15%)	0.86

Surgery type: thoracic *n *(%)	4 (6%)	5 (7%)	0.75

Surgery type: abdominal *n *(%)	37 (54%)	36 (52%)	0.92

Surgery type: ear, nose, and throat *n *(%)	4 (6%)	6 (9%)	0.53

Surgery type: orthopedic lower extremity *n *(%)	9 (13%)	4 (6%)	0.24

Surgery type: obstetrics and gynecology *n *(%)	13 (19%)	14 (20%)	0.83

Surgery type: orthopedic upper extremity *n *(%)	2 (3%)	3 (4%)	0.68

Surgery type: orthopedic spine *n *(%)	1 (1%)	1 (1%)	0.98

Admission diagnosis: trauma *n *(%)	15 (15%)	17 (18%)	0.68

Pre-unit length of stay, mean (SD) days	3.57 (5.58)	4.20 (5.89)	0.45

TISS-28 score day 1, mean (SD)	32.23 (5.21)	31.22 (6.12)	0.29

Infective criteria: (1) Temperature >38^0^C or <36^0^C; (2) white blood count >12 × 10^9^/L or <4 × 10^9^/L; (3) pus present in body cavity or tissues.

### Clinical outcomes

#### Ventilation

Fifty-two percent (100 of 193) of the sample received ventilation on unit admission. Patients admitted to the unit during protocol care were less likely to be intubated after unit admission than were patients admitted during usual care (RR, 0.16; 95%CI, 0.07 to 0.71; RRR, 0.84; NNT, 5.02; *P *= 0.005).

Seventeen extubations of 14 patients failed during the two intervention periods. Three patients were reintubated twice. During usual care, 14 of 68 attempted extubations failed; and three of 47, during protocol care. The risk of failing an extubation was 77% less when the patient was admitted to the unit during the protocol-care intervention period compared with the usual-care intervention period (RR, 0.23; 95% CI, 0.05 to 0.98; RRR, 0.77; NNT, 6.95; *P *= 0.04). The mean difference in the ventilation time of patients admitted during usual care was 5.10 hours (95% CI, 9.65 to 19.84) when compared with the protocol-care condition periods. This difference was not significant (*p *= 0.50).

#### Mortality

Seventeen (9%) patients died. Twelve (70%) patients died in the unit, whereas the remaining five (30%) patients died in the hospital. No difference in mortality was found between groups (*p *= 0.52).

### Economic outcomes

#### Length of stay

No difference was noted in unit LOS. However, protocol-care patients were discharged from the hospital 4 days earlier than usual-care patients. This potential clinically important difference did not reach statistical significance in this sample (*p *= 0.05; Table 4).

**Table 4:** Length of stay

	Usual care	Protocol care	Mean difference meters (95% CI)	*P*
Hospital length of stay Days (mean ± SD)	17.13 ± 14.38	14.47 ± 11.00	2.65 (-1.86 to 7.17)	0.20

ICU length of stay Days (mean ± SD)	71.80 ± 48.51	71.61 ± 61.82	0.19 (-1.20 to 1.22)	0.98

Time after unit discharge Days (mean ± SD)	10.50 ± 11.68	7.41 ± 7.45	3.97 (-0.35 to 6.5)	0.05^a^

^a^Tendency toward significance. CI, confidence Interval; SD, standard deviation.

#### TISS-28

**Figure 3:**
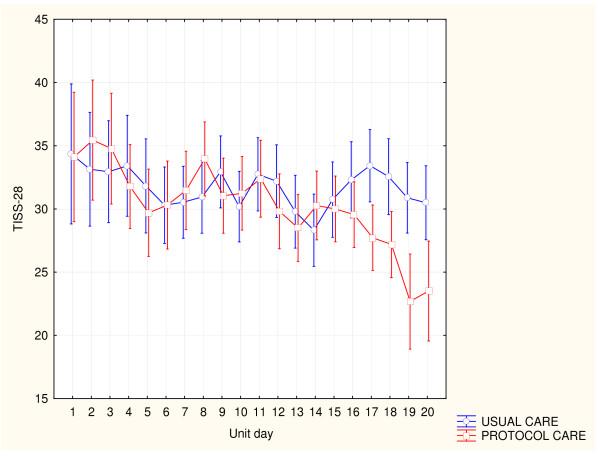
**Change in TISS-28 day score during condition periods**.

Eighty percent of the sample (*n *= 154) were managed in the unit for the duration of a TISS-28 unit day, resulting in 499 TISS-28 scores. No difference was found in the TISS-28 scores on unit admission (Table 3). A change in the mean unit TISS-28 score was observed over time during both condition periods (Figure 3). This decrease was greater during the protocol-care condition period (*p *= 0.04) and more distinct over the final week of the protocol-condition periods. The mean difference in the cumulative daily unit TISS-28 score during the two condition periods was 1.99; 95% CI, 0.65 to 3.35; TISS-28 units (*p *= 0.04). The mean difference in individual patient's daily TISS-28 score during the two condition periods was 1.92 (95% CI, 0.11 to 3.95; *P *= 0.06). The burden of nursing care was significantly different in six of 27 TISS-28 categories when condition periods were compared (Table 5).

**Table 5:** The proportion of the daily TISS-28 items for which the activity was recorded

	Usualcare *n *= 254	Protocol care *n *= 245	*P*
Standard monitoring: hourly vital signs, regular registration, and calculation of fluid balance	100%	100%	1.00

Laboratory: biochemical and microbiological investigations	100%	100%	1.00

Single medication: intravenously, intramuscularly, subcutaneously, and/or orally (for example, gastric tube)	1.96%	0.10%	0.38

Multiple intravenous medication: more than one drug, single shots or continuously	97.63%	97.95%	1.00

Routine dressing changes: care and prevention of decubitus, daily dressing change	99.21%	99.18%	1.00

Frequent dressing changes: frequent dressing change (at least one time in each nursing shift) and/or extensive wound care	99.60%	99.18%	1.00

Care of drains: all (except gastric tube)	64.97%	49.38%	<0.000^a^

Mechanical ventilation: any form of mechanical ventilation/assisted ventilation with or without PEEP, with or without muscle relaxants; spontaneous breathing with PEEP	74.80%	54.69%	<0.000^a^

Supplementary ventilatory support: breathing spontaneously through endotracheal tube without PEEP; supplementary oxygen (any method)	25.59%	45.71%	<0.000^a^

Care of artificial airways: endotracheal tube or tracheostomy	66.93%	56.73%	0.02^a^

Single vasoactive medication: any vasoactive drug	23.62%	22.04%	0.67

Multiple vasoactive medications: more than one vasoactive drug, disregarded type and dose	7.87%	5.31%	0.24

IV replacement of large fluid losses: fluid administration 3 L/m^2^/day, disregarding type of fluid administered	58.66%	45.71%	0.004^a^

Peripheral artery line	97.64%	97.55%	0.95

Left atrium monitoring: Swan-Ganz catheter with or without cardiac-output measurement	1.18%	5.71%	0.005^a^

Central venous line	91.73%	88.98%	0.30

Cardiopulmonary resuscitation after arrest: in the past 24 hours (single precordial percussion not included)	1.96%	0.82%	0.27

Renal support: hemofiltration techniques	3.15%	2.86%	0.85

Renal support: quantitative urinary-output measurement	99.21%	99.59%	0.58

Renal support: active diuresis	30.71%	28.98%	0.67

Neurologic support: measurement intracranial pressure	1.0%	0	0.25

Treatment of complicated metabolic acidosis/alkalosis	0.79%	0	0.16

Intravenous hyperalimentation	4.33%	3.27%	0.54

Enteral feeding: through gastric tube or other GI route (for example, jejunostomy)	38.19%	37.55%	0.88

Single specific intervention in the ICU: such as naso- or orotracheal intubation, introduction of pacemaker, cardioversion, endoscopies, emergency surgery in the past 24 hours, gastric lavage, CVP; Swan-Ganz; dialysis catheter Routine interventions without direct consequences to the clinical condition of the patient are not included, such as x-rays, echography, ECG, dressings, introduction of peripheral lines	11.81%	15.92%	0.18

Multiple specific interventions in the ICU: more than one, as described above	3.93%	1.23%	0.08^b^

Specific interventions outside the ICU: (for example, surgery or diagnostic procedures for which patient must leave unit (CT; bronchoscopy)	10.63%	6.54%	0.10

^a^Significant. ^b^Tendency toward significance. CT, computed tomography; CVP, central venous pressure; ECG, electrocardiogram; GI, gastrointestinal; PEEP, positive end-expiratory pressure.

### Patient-centered outcome

#### Barthel Index

Ninety patients completed the Barthel Index (Table 6). A tendency was noted for a greater proportion of patients who received protocol care to reach independence in transfer (*p *= 0.07) and mobility categories (*p *= 0.09). This did not reach statistical significance in this sample (Table 7).

**Table 6:** Summary of reasons patients were not included in Barthel Index

Patient	Usual care *n *= 96	Protocol care *n *= 95	*P *value
Excluded	40	40	0.95
No consent	7	6	0.78
Died in unit	5	4	0.75
In unit <24 hours	7	10	0.61
Premorbid state	0	1	0.50
Patients discharged during washout period	21	19	0.76
Lost to follow-up	12	9	0.49
Patient died in hospital before test	2	0	0.25
Discharged before test	6	9	0.82
Patient readmitted to unit	1	0	0.5
Missing data	3	0	0.12

Patients were excluded from functional testing when admitted to the unit <24 hours; discharged from ICU >48 hours; not orientated to time and place; presented with head injuries/spinal cord injuries/unstable pelvis and vertebral fractures; preadmission history of dementia/confusion, Alzheimer disease; refused to provide informed consent; dependent before unit admission; orthopedic or vascular contraindications to mobilization.

**Table 7:** Proportion of patients who reached independence in the Barthel Index category

	Usual care *n *= 44	Protocol care *n *= 46	*P*
Feeding	61%	67%	0.28
Bathing	80%	85%	0.25
Grooming	91%	89%	0.78
Dressing	32%	35%	0.53
Bowels	39%	48%	0.21
Bladder	57%	70%	0.21
Toilet	59%	63%	0.65
Transfers	68%	83%	0.07^a^
Mobility	50%	67%	0.09 ^a^
Stairs	3%	9%	0.18

^a^Tendency toward significance.

## Discussion

The findings of this study are the first to support the notion that the method of delivering physiotherapy care can affect patient outcomes [41]. The service-delivery method consisting of an exclusively allocated physiotherapist guided by a validated evidence-based protocol improved patient outcomes. Protocol care decreased the number of intubations, increased successful extubations, and accelerated the decrease in unit TISS-28 score. In addition, a greater proportion of patients reached independence in the transfer and mobility categories of the BI. The observed difference in the functional ability did not reach statistical significance in this sample. Whether this clinically important observed difference was just by chance will have to be investigated in a sufficiently powered study. The two components of the service that differed from usual care include the physiotherapist availability and the use of an evidence-based physiotherapy protocol.

The availability of an exclusively allocated therapist resulted in shorter waiting periods for physiotherapy and ensured physiotherapy care to all patients in the unit compared with usual care. Usual-care therapists were at liberty to decide on the duration and frequency of therapy, but organizational barriers could have restricted more-frequent sessions, although indicated. More frequent and earlier exposure to physiotherapy care could have reduced the need for intubation [42,43].

Even though the techniques documented during the two condition periods were similar, the frequency of technique use differed significantly. Therapeutic content of sessions documented during protocol-care condition periods were aligned with the internationally agreed-on evidence-based protocol [26]. The protocol was not prescriptive. The hierarchic framework provided clinicians with best practice-management options to consider. Differences noted in technique selection could be indicative of the decision-making process used during the two condition periods. This does not negate the potential that specific patients managed during usual care received evidence-based care.

The average number of sessions provided during protocol care (1.5 sessions/patient/ICU day) compared favorably with published reports [44]. This may allude to the fact that simply increasing the number of physiotherapy sessions would not necessarily account for the improved patient outcomes. Providing six respiratory physiotherapy sessions per day to mechanical ventilated (>24 hours) acute-brain-injury patients did not reduce the incidence of ventilator-associated pneumonia, ventilation time, or ICU/hospital LOS compared with standard nursing care [45]. To assess cost effectiveness, it will be necessary to compare the outcomes of patients from surgical units in which physiotherapy is not provided with the service approaches documented here.

We used the TISS-28 instrument to measure the impact on cost on two physiotherapy-service approaches to care. Based on the mean difference in the unit TISS-28 score between the two intervention periods, we calculated a saving of 3,334.85 Euros over the 6- week protocol-care period [35]. The cost of research therapists' salaries during the protocol-care period was ±2,848 Euros (R32,000). The difference in the observed mean TISS-28 score is related to a decrease in reported nursing activity observed in the ventilation and chest-drain categories during protocol care. This finding is confirmed by the differences in service approach. Shorter waiting periods for physiotherapy and increased mobility have been linked to early removal of chest drains [46,47]. Patients' inability to manage excessive secretions has been linked to failed extubations [48]. Deep-breathing exercises and increased mobility reported during protocol care are regarded as successful secretion-management strategies and could explain the difference noted in the documented nursing activities in the mechanical-ventilation category during protocol care. Mechanical ventilation is associated with significantly higher cost of ICU care [49]. In addition, the potential savings of earlier hospital discharge and the potential decreased burden of post-ICU rehabilitation must be considered. Even though we observed a greater proportion of protocol-care patients reaching independence in BI categories, this did not reach statistical significance in this sample. However, the observation of a greater proportion of patients reaching functional independence after early mobility is supported by other articles reporting on improved functional ability in patients after early ICU mobility [11,50]. A decrease in the need for post-ICU rehabilitation would further decrease cost and thus strengthen the argument for the cost benefit of service, which includes an exclusively allocated physiotherapist providing evidence-based/protocol care when compared with usual care.

Although we offer limited data on the potential savings of offering protocol care, we do not provide data on the potential impact on work-related therapist injuries. This must be explored in future studies.

The trial design provides potential for bias. Randomization of condition options, usual care, and protocol care, was problematic because of the interdisciplinary nature of the protocol. It would be difficult not to have dilution of usual care if it were to be provided simultaneous with protocol care. Although the randomization of intervention periods could be a novel way in which two services could be compared in a single unit, future studies should rather consider a cluster randomization of centers in an international multicenter randomized controlled trial (RCT). Because it was not possible to randomize patients to the intervention options, the possibility that patients were different at baseline cannot be excluded. However, decisions regarding unit admission were made by the unit director based on institutional criteria. The physiotherapy service provided at a specific time would not have influenced the decision. The trial periods were also interspersed over a relatively short period, negating possible population bias, which could affect baseline characteristics (for example, the H1N1 flu pandemic.

We acknowledge that the results of this trial could be due to the increased volume/availability of therapy delivered: a combination of the increased volume/availability of therapy and the application of treatment protocols or the treatment protocols alone. The aim of this study was to compare two physiotherapy-service approaches. To facilitate clinical application, future studies should discern between these components.

These results must be interpreted with caution. This pragmatic trial was designed to explore the preliminary effect of a dedicated evidence-based physiotherapy/protocol service on a variety of outcomes. A range of outcomes was included to reflect all aspects of the service provided [51]. These results should be affirmed in a sufficiently powered multicenter RCT.

## Conclusions

A physiotherapy-service approach that includes an exclusively allocated physiotherapist providing evidence-based/protocol care that addresses pulmonary dysfunction and promotes early mobility improves patient outcome. This approach decreased the number of intubations and reintubations and decreased the burden on nursing care. This could be a more cost-effective service approach to care than usual care. This information can now be considered by administrators in the management of scarce physiotherapy resources and by researchers in the planning of a multicenter RCT.

## Key messages

• The method of delivering physiotherapy care can affect patient outcomes.

• The TISS-28 is sensitive to measure a change in the physiotherapy-service approach to care provided in a surgical ICU.

• A physiotherapy-service approach that includes an exclusively allocated physiotherapist providing evidence-based/protocol care decreases intubation and reintubation rates in a surgical ICU.

• This could be a more cost-effective service approach to care than usual care.

## Abbreviations

APACHE II: Acute Physiologic and Chronic Health Evaluation II; BI: Barthel Index; CVP: central venous pressure; CT: computed tomography; CI: confidence interval; DBE: deep-breathing exercise; ECG: electrocardiogram; GI: gastrointestinal; ICU: intensive care unit; IPPB: intermittent positive-pressure breathing; LOS: length of stay; NNT: number needed to treat; PI: principal investigator; PEP: positive expiratory pressure; PEEP: positive end-expiratory pressure; RCT: randomized controlled trial; RR: relative risk; RRR: relative risk reduction; SD: standard deviation; TISS-28: Therapeutic Intervention Scoring System 28 item.

## Competing interests

The authors declare that they have no competing interests.

## Authors' contributions

SH conceived of the study; participated in the design of the study; was responsible for training of research therapists and data collection; performed statistical analysis, interpreted data; and helped to draft the manuscript. QL participated in the design of the study; performed statistical analysis and interpreted data; and helped to draft the manuscript. AC participated in the design of the study and interpreted data. All authors read and approved the final manuscript.
